# Comparison of the efficacy and safety of MAKO robot-assisted total knee arthroplasty versus conventional manual total knee arthroplasty in uncomplicated unilateral total knee arthroplasty a single-centre retrospective analysis

**DOI:** 10.1007/s00264-024-06234-0

**Published:** 2024-06-14

**Authors:** Nanshan Ma, Pengfei Sun, Pengfei Xin, Sheng Zhong, Jun Xie, Lianbo Xiao

**Affiliations:** 1 Shanghai University of Traditional Chinese Medicine, Shanghai, People’s Republic of China; 2 Department of Orthopedic Surgery, Shanghai Guanghua Hospital of Integrative Medicine, Shanghai, People’s Republic of China

**Keywords:** Robot-Assisted, MAKO, Total Knee Arthroplasty, Efficacy, Safety

## Abstract

**Purpose:**

To compare the efficacy and safety of MAKO robot-assisted total knee arthroplasty (MA-TKA) with conventional manual total knee arthroplasty (CM-TKA) in patients with end-stage knee osteoarthritis (KOA) during the early postoperative period.

**Method:**

A retrospective analysis was conducted on 22 patients with KOA who underwent MA-TKA and 26 patients who underwent CM-TKA from April 2023 to July 2023. Hip-knee-ankle angle (HKA), lateral distal femoral angle (LDFA), medial proximal tibial angle (MPTA), American Knee Society Score (AKSS), Forgotten Joint Score-12 (FJS-12), visual analogue scale (VAS), and postoperative complications were recorded and compared between the two groups.

**Result:**

Both groups successfully completed the surgeries. In terms of radiographic parameters, postoperative one month LDFA and HKA in the MA-TKA group were significantly lower than those in the CM-TKA group (*P* < 0.05). At the one month follow-up, 19 patients (86.4%) in the MA-TKA group had an HKA less than 3°, compared to 20 patients (76.9%) in the CM-TKA group. Clinically, VAS scores at 24 h, 48 h, and 72 h postoperatively were lower in the MA-TKA group both at rest and during activity. At one month and three months postoperatively, AKSS Function Scores and FJS-12 scores in the MA-TKA group were significantly higher than those in the CM-TKA group (*P* < 0.05). Regarding postoperative complications, no complications occurred in the MA-TKA group, while one patient in the CM-TKA group experienced postoperative knee stiffness, which resolved after physical therapy, with no statistically significant difference (*P* > 0.05).

**Conclusion:**

Compared with conventional manual total knee arthroplasty, MAKO robot-assisted TKA demonstrates better short-term clinical efficacy, achieves better alignment planning, and maintains good safety.

## Introduction

As one of the most successful surgeries of the last century, total knee arthroplasty (TKA) has become one of the most common surgical options for patients with severe knee osteoarthritis who have failed conservative treatment [1, 2]. An epidemiological study showed that from 2013 to 2018, a total of 4503 patients underwent revision TKA surgeries registered in the Chinese Hospital Quality Monitoring System, accounting for only 2.4% of all patients who underwent TKA surgeries during that period [3]. TKA involves reshaping the dysfunctional joint through osteotomy and placing specific artificial joint prostheses, which can restore normal function to the knee joint in patients with end-stage osteoarthritis, alleviate pain, and improve knee joint mobility.

Traditional TKA surgeries are mostly performed by orthopaedic surgeons using cutting guides for osteotomy, and the placement position and angle of the cutting guides during surgery depend on the surgeon's standard operation and surgical experience. Failure to achieve precise osteotomy or appropriate soft tissue balancing can significantly affect the stability of the postoperative coronal alignment and prosthetic positioning, increasing the rate of prosthetic wear and the need for revision surgeries [4, 5, 6]. The MAKO surgical robot (Stryker, Kalamazoo, Michigan, USA) as the latest robotic-assisted joint replacement technology, assists orthopedic surgeons in more accurately performing osteotomy and balancing ligament tension during surgery through preoperative analysis of the patient's three-dimensional CT scans, thereby achieving more satisfactory surgical outcomes [7, 8]. Currently, the MAKO surgical robot can not only be used to guide intraoperative correction of severe knee varus deformities but also participate in intraoperative planning for surgeries such as total hip arthroplasty (THA) and revision TKA, and it has been widely used in orthopedic surgeries worldwide [9–12]. This study aims to compare the early clinical efficacy of TKA assisted by the MAKO robot and conventional manual total knee arthroplasty in the treatment of patients with end-stage knee osteoarthritis through retrospective analysis.

## Materials and methods

### Patient data

A retrospective analysis was conducted on 48 patients who underwent total knee arthroplasty from April 2023 to July 2023. Among them, 22 patients underwent total knee arthroplasty under the guidance of the MAKO robotic navigation system (MA-TKA Group), while 26 patients underwent conventional manual total knee arthroplasty (CM-TKA Group). All surgeries are performed by the same lead surgeon.

### Inclusion and exclusion criteria

Inclusion Criteria: 1. Diagnosis of end-stage knee osteoarthritis, with Kellgren-Lawrence grade III or IV, ineffective conservative treatment, and clear surgical indications; 2. All patients were undergoing unilateral knee surgery for the first time; 3. No contraindications for surgery, informed about the treatment plan, and signed informed consent.

Exclusion Criteria: 1. Patients with neurological or musculoskeletal disorders, preoperative knee joint infections, or knee joint fractures; 2. Patients with severe knee joint instability who cannot use standard prostheses with preserved collateral ligaments and require restrictive prostheses or extension rods; 3. Patients with severe postoperative complications, such as local haematoma, deep vein thrombosis, requiring secondary surgery; 4. Patients with incomplete clinical and radiographic follow-up data.

### Surgical and perioperative protocols

CM-TKA Group patients underwent conventional total knee arthroplasty. After successful anaesthesia, the patient was placed in the supine position, and a tourniquet was applied following routine disinfection and draping. A median incision was made in front of the knee, and the skin, subcutaneous tissue, and fascia were incised layer by layer. The medial parapatellar approach was used to open the joint capsule, externally rotate the patella, and flex the knee to expose the joint cavity. The synovial membrane, medial and lateral menisci, and cruciate ligaments were resected, along with any bony prominences of the femur and tibia. Standard osteotomies of the tibia and femur were performed with the assistance of osteotomy guides. The hypertrophic ligaments were excised, joint deformity corrected, and a trial implant inserted. After confirming proper patellofemoral tracking with the "no-thumb test," bone cement was prepared, and the prosthesis was implanted. Excess cement was removed, and the area was irrigated with povidone-iodine solution for 5 min to prevent infection. After ensuring haemostasis the wound was closed in layers, and local infiltration anaesthesia was administered before closure. Clean dressings and compression bandages were applied, and elastic bandages were used to prevent deep vein thrombosis.

Patients in the MA-TKA Group underwent preoperative CT scans of the affected hip, knee, and ankle joints, which were imported into the MAKO robotic system. A three-dimensional model of the knee joint was created, and during surgery, fixed tibial and femoral navigation frames were installed. After resecting the menisci and cruciate ligaments, the femur and tibia were registered, and osteotomies were performed according to the preoperative plan using the robotic arm. The remaining procedures followed traditional TKA techniques. Both groups received multimodal postoperative pain management, including self-controlled intravenous analgesia for the first 48 h. Oral non-steroidal anti-inflammatory drugs or weak opioids were administered if necessary. Rivaroxaban 10 mg qn was given orally for postoperative anticoagulation, starting on the evening of surgery and continuing for four weeks. Additionally, cefazolin sodium 1 g was infused intravenously on the day of surgery for infection prophylaxis, with dosage adjustments based on postoperative blood reexamination results. Both groups underwent continuous passive motion (CPM) and isokinetic plyometric training, along with postoperative knee joint functional exercises under the guidance of the same rehabilitation therapist.

### Measurement of follow-up outcome indicators

**Fig. 1:**
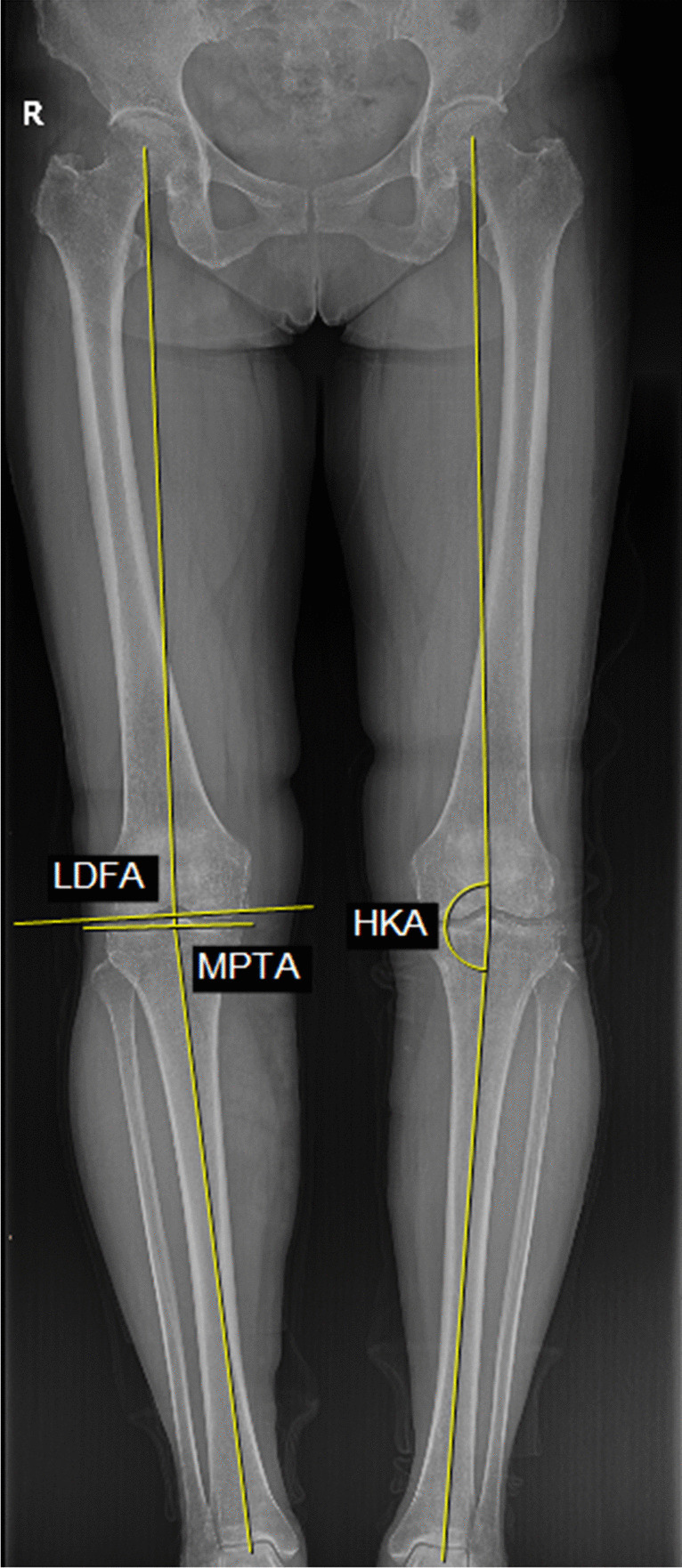
**HKA:** The angle formed by the intersection of the lines connecting the centre of the femoral head to the center of the knee joint and the center of the knee joint to the centre of the ankle joint (record the supplementary angle of this angle). **LDFA:** The angle formed between the medial and lateral tangent lines of the femur and the mechanical axis of the femur. **MPTA:** The angle formed between the medial and lateral tangent lines of the tibia and the mechanical axis of the tibia

Radiographic outcome evaluation: All patients underwent knee joint anteroposterior and lateral X-ray examinations preoperative and at one month postoperatively. We used the medial proximal tibial angle (MPTA), lateral distal femoral angle (LDFA), and hip-knee-ankle angle (HKA) as measurement indicators. MPTA, LDFA, and HKA are important radiographic indicators commonly used to evaluate knee joint morphology, diagnose KOA, and assess the corrective effects of TKA postoperatively. The measurement methods are shown in Fig. 1.

Clinical outcome evaluation: Pain intensity and activity recovery in the early postoperative period were assessed by recording the Visual Analog Scale (VAS) scores at 24 h, 48 h, and 72 h postoperatively, as well as the American Knee Society Score (AKSS) and Forgotten Joint Score-12 (FJS-12) at one, three and six months postoperatively for all patients. The AKSS score is divided into two parts, the first part is a clinical assessment of the knee through physical examination and the second part is an assessment of the individual's functioning, both of which are scored out of a total of 100 points. The AKSS is very useful and reliable for evaluating patients with osteoarthritis or those undergoing TKA [13]. The FJS-12, a new scoring method, measures a patient's ability to forget the artificial joint in daily life. The FJS-12 score can better help clinicians assess and track the progress of patients' rehabilitation after TKA, and is now widely used [14]. Concurrently, complications occurring after surgery in all patients were also documented .

### Statistical analysis methods

All data were analyzed using SPSS 26 statistical software (IBM Corporation, USA). The Shapiro–Wilk test was used to assess whether variables followed a normal distribution. Normally distributed continuous data are expressed as mean ± standard deviation (SD), whereas non-normally distributed continuous data are presented as median (interquartile range). Independent sample t-tests were employed to compare normally distributed continuous data between groups. The Mann–Whitney U test was utilized for non-normally distributed continuous data. Group comparisons of categorical variables were performed using the chi-square test. In this study, *P* < 0.05 was considered statistically significant.

## Results

### General results

A total of 48 patients (18 males and 30 females) were included in this study, with a mean age of 67.31 ± 7.25 years. All 48 patients underwent successful surgery without intraoperative blood transfusion. The length of hospital stay ranged from five to 14 days, with a mean of 9.63 ± 2.7 days. The surgical time ranged from 56 to 151 min, with a mean of 105.4 ± 29.01 min. Among them, 26 patients underwent conventional manual total knee arthroplasty (CM-TKA Group), while 22 patients underwent total knee arthroplasty (MA-TKA Group) with the MAKO robotic navigation device. Table 1 displays the characteristics of the two patient groups. No statistically significant differences were observed in the baseline characteristics between the two patient groups except for the surgical time.

**Table 1:** Characteristics of included patients

	MA-TKA Group (*n* = 22)	CM-TKA Group (*n* = 26)
Age	68.68 ± 7.92	66.15 ± 6.56
Gender		
Male	7(31.8%)	11(42.3%)
Female	15(68.2%)	15(57.7%)
Surgical Site		
Left Knee	13(59.1%)	12(46.2%)
Right Knee	9(40.9%)	14(53.8%)
K-L Grade		
III	2(9.1%)	1(3.85%)
IV	20(90.9%)	25(96.15%)
HKA (preoperative)	6.67 ± 3.29°	6.53 ± 3.42°
Surgical time(min)	133.55 ± 9.17	81.58 ± 14.99*
MPTA(preoperative)	84.93 ± 2.42	83.77 ± 2.21
LDFA(preoperative)	85.04 ± 2.35	86.03 ± 2.32

*: *P* < 0.05

### Comparison of the postoperative follow-up results of TKA in the two groups of patients

**Fig. 2:**
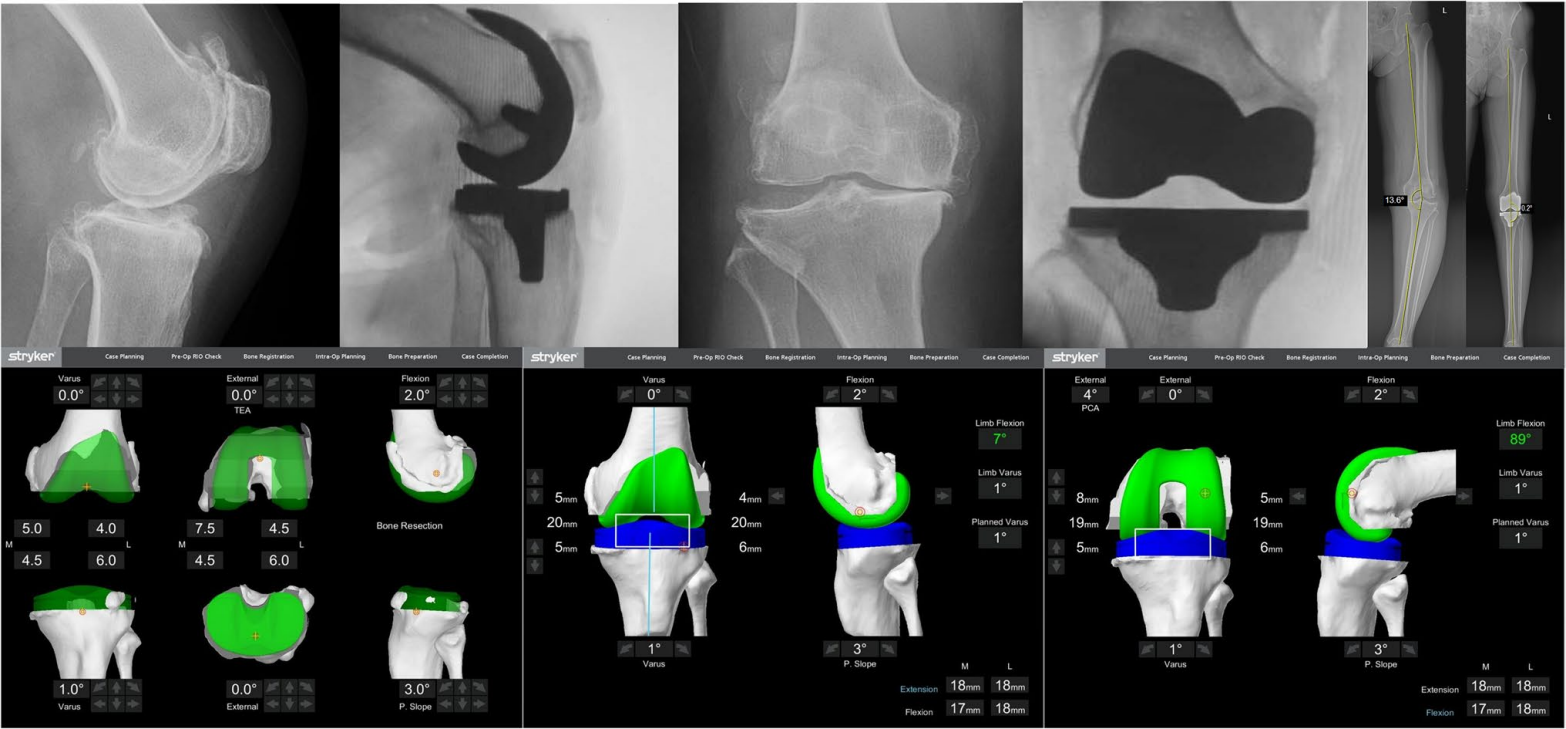
**Typical Case:** Female, 67 years old, left knee osteoarthritis, underwent MAKO robotic-assisted TKA surgery. Preoperative X-ray examination revealed narrowing of the medial tibiofemoral joint space, osteophyte formation, lip-like changes, and soft tissue swelling around the joint. The preoperative HKA is approximately 13.6°. Following surgery, the patient achieved balanced knee joint gaps in both extension and flexion states according to the preoperative osteotomy plan. Postoperative X-ray examination showed changes after left knee arthroplasty, with a well-maintained joint space and proper positioning of the prosthesis. The postoperative HKA is approximately 0.2°

In terms of radiographic outcomes, at the last follow-up, the LDFA and HKA in the MA-TKA group were all lower than those in the CM-TKA group (*P* < 0.05). The radiographic outcomes at the last follow-up between the two groups are presented in Table 2. At the last follow-up, 86.4% of MA-TKA Group patients had an HKA less than 3°, while 76.9% of CM-TKA Group patients had an HKA less than 3° (Fig. 2).

**Table 2:** Comparison of Radiographic Examination Indicators at 1 Month Follow-up

	MA-TKA Group (*n* = 22)	CM-TKA Group (*n* = 26)
MPTA	89.87 ± 0.75°	90.41 ± 1.11°
LDFA	91.05 ± 0.82°	92.64(90.83, 93.13) °*
HKA	1.86 ± 1.03°	2.63 ± 0.66°*

*: *P* < 0.05

**Fig. 3:**
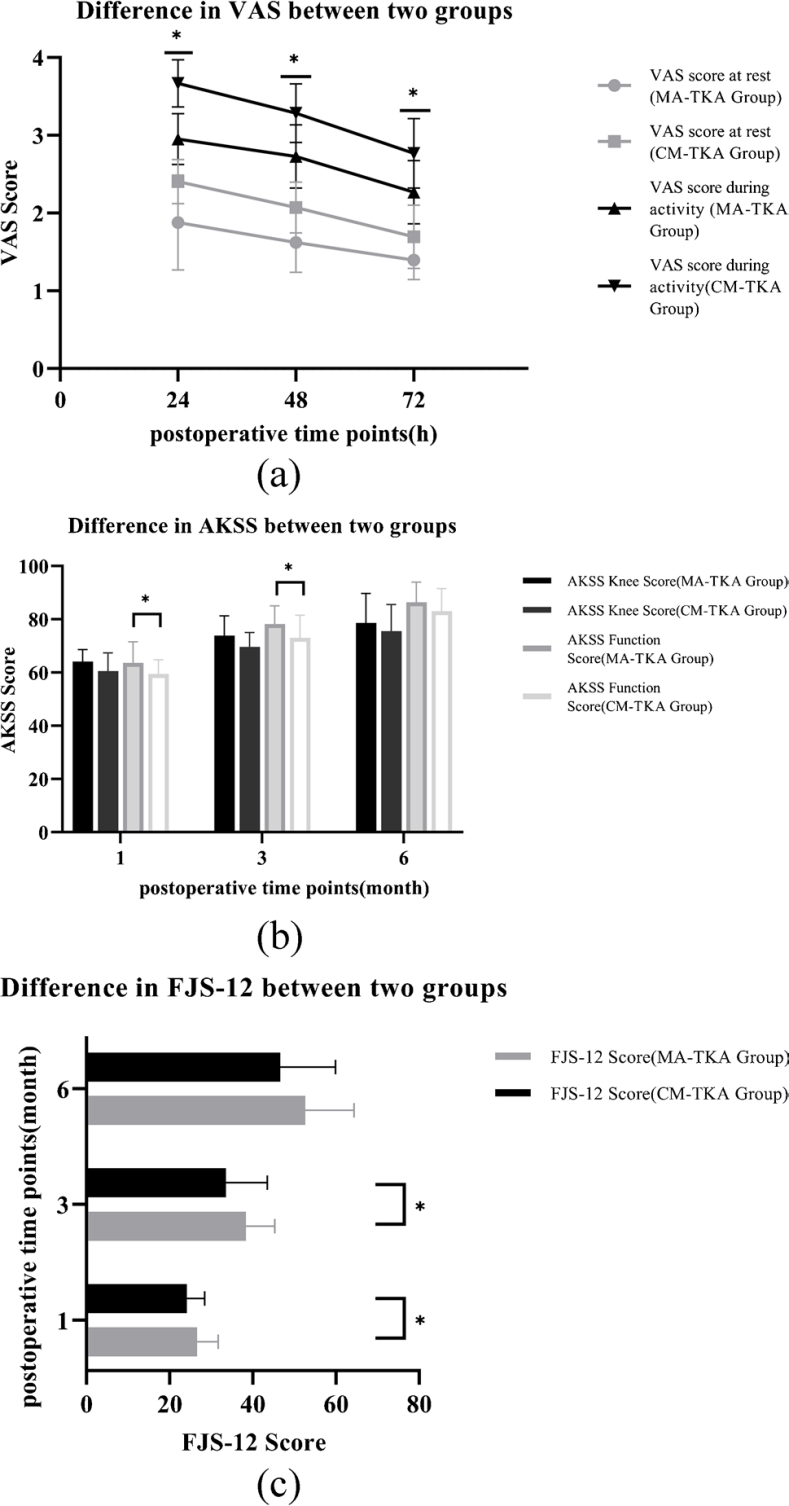
Comparison of Clinical Indicators in Follow-up after TKA in Two Groups. (a: Comparison of VAS scores between the two groups after surgery, b: Comparison of AKSS scores between the two groups after surgery, c: Comparison of FJS-12 scores between the two groups after surgery. *P < 0.05)

In terms of clinical outcomes, at 24 h, 48 h, and 72 h postoperatively, VAS scores in the MA-TKA group were lower than those in the CM-TKA group both at rest and during activity. The differences were statistically significant (*P* < 0.05). At one month and three months postoperatively, AKSS Function Score and FJS-12 scores in the MA-TKA group were higher than those in the CM-TKA group, with statistically significant differences (*P* < 0.05). The comparison of AKSS and FJS-12 scores between the two groups at the remaining follow-up time points was not statistically significant, as shown in Fig. 3. Regarding postoperative complications, no complications occurred in the MA-TKA group, while one patient in the CM-TKA group experienced knee joint stiffness postoperatively, which recovered after physical therapy. There was no statistically significant difference in postoperative complications between the two groups (*P* > 0.05).

## Discussion

In recent years, there has been rapid development in surgical robots, leading to the integration of robotics into joint replacement surgery. The MAKO robot is currently the most widely used and intelligent surgical robot for joint procedures worldwide. It assists surgeons in completing a wide range of surgeries, including TKA, THA, and others. In TKA surgery, accurate prosthesis placement position and angle are essential to ensure the normal lower limb biomechanics after surgery. JEFFERY et al. [15] analyzed the postoperative imaging data of 115 patients who underwent total knee replacement and found that the prosthesis loosening rate was 24% when the mechanical axis deviated by more than ± 3°, whereas the loosening rate for patients with the mechanical axis within ± 3° was only 3%. This indicates that precise alignment is crucial for successful outcomes. The MAKO robot can help KOA patients restore a more reasonable lower limb biomechanics by guaranteeing precise knee joint alignment through accurate preoperative 3D CT image measurements [16]. Meanwhile, the MAKO robot can assist in achieving preoperatively planned bone resection during surgery, avoiding secondary bone recutting caused by inappropriate bone resection, and is a reliable method for predicting and achieving knee joint balance [17–21].

KEHLET first proposed the concept of Enhanced Recovery After Surgery (ERAS) in 1993, which aims to improve postoperative recovery by reducing perioperative stress responses and pain. TKA, as the primary means of treating end-stage knee osteoarthritis, achieves a patient satisfaction rate of 87% in the first year postoperatively [22, 23]. The core of Enhanced Recovery After Surgery in TKA is pain management. The MAKO robot can help reduce postoperative pain and accelerate postoperative knee joint function recovery. Batailler et al. conducted a systematic review of 26 studies involving MAKO robot-assisted TKA surgeries, which showed that compared to traditional TKA, MAKO robot-assisted TKA could alleviate postoperative pain and significantly improve prosthetic positioning [24]. Additionally, several studies have shown that patients undergoing MAKO robot-assisted TKA surgeries have better early systemic inflammatory responses than those undergoing traditional TKA surgeries [25–27]. In terms of safety, MAKO robot-assisted TKA has been shown to reduce damage to the soft tissues around the knee joint [28]. Kayani et al. found in a prospective cohort study that compared to traditional total knee arthroplasty, robot-assisted TKA resulted in less bone and soft tissue damage around the joint [29]. Similarly, cadaveric studies have shown similar findings, with robot-assisted TKA resulting in less damage to the soft tissues around the knee joint, especially the posterior cruciate ligament [30], compared to traditional total knee arthroplasty. With the assistance of the MAKO robot, the range and amount of bone resection are predetermined preoperatively. When the system detects bone resection exceeding the planned amount or the saw blade tip approaching the safe boundary of bone resection, the saw blade automatically shuts off, preventing further bone resection. Additionally, ligaments and soft tissues around the knee joint are set outside the operating area, significantly reducing the risk of damaging the medial and lateral collateral ligaments, the hamstring tendon, and the soft tissues around the knee joint [31]. Regarding the learning curve, studies have shown that the learning curve for robot-assisted total knee arthroplasty ranges from 6 to 20 cases, with a significant reduction in total surgical length after the 30th case. The experience of using the MAKO robot does not affect implant positioning, preoperative planning, and postoperative complications. After the learning curve, there was no significant difference in surgical time between robot-assisted TKA and traditional total knee arthroplasty, and total surgical time could be significantly reduced early in the learning phase [32–34].

In this study, through retrospective analysis, we found that the postoperative HKA angle and LDFA angle in the MA-TKA Group were lower than those in patients receiving traditional manual total knee arthroplasty, and the proportion of patients with the HKA angle less than 3° after surgery was higher than that in the CM-TKA Group. The MAKO robot, by analyzing preoperative CT images of patients, generates and guides reasonable bone resection schemes during surgery, helping patients achieve better prosthetic placement and lower limb alignment after surgery, thereby achieving more accurate knee joint alignment, ensuring postoperative knee joint stability, and avoiding postoperative knee joint stiffness or adhesion due to loosening of the prosthesis or padding. We also noticed that at 24 h, 48 h, and 72 h postoperatively, VAS scores of patients in the MA-TKA Group were lower than those in the CM-TKA Group, and AKSS Function Scores and FJS-12 scores of patients in the MA-TKA Group were higher than those in the CM-TKA Group at 1 month and 3 months postoperatively. We believe that, on the one hand, as mentioned earlier, the MAKO robot can adjust the prosthetic position in real-time during surgery, achieve intraoperative compartmental balance, stabilize lower limb alignment, and enable patients to gain better mobility in the short term after surgery. On the other hand, traumatic damage to the knee joint capsule during knee joint replacement surgery can lead to postoperative capsule hypertrophy and contraction, and scar tissue proliferation inside the knee joint postoperatively can also result in stiffness and pain during knee joint motion. The precise navigation of the MAKO robot during surgery can reduce excessive damage to the soft tissues around the knee joint, not only helping alleviate the pain caused by the postoperative short-term joint capsule soft tissue contraction but also indirectly reducing postoperative scar tissue proliferation, increasing patients' short-term mobility after surgery, and improving postoperative satisfaction. In terms of safety, the precise navigation of the MAKO robot during surgery can avoid excessive bone resection, and the automatic power-off mechanism of the saw blade ensures that the important blood vessels and nerves around the knee joint are not damaged during surgery, greatly reducing the surgical risk. The average operation time for patients in the MA-Group was 133.55 ± 9.17 min, higher than that for patients in the CM-TKA Group, which was 81.58 ± 14.99 min, which may be partly attributed to the learning curve associated with the new technology. For the surgical time of MA-TKA Group, our study showed that the first six surgeries took more than 140 min with a mean surgical time of 144.5 ± 3.73 min, while the surgical time after the 15th surgery was within 130 min with a mean of 122.86 ± 4.26 min, indicating that MAKO robotic-assisted TKA surgery has a significant learning curve. Additionally, the operation time for TKA surgeries assisted by the MAKO robot decreased as surgical volume increased in our research team, eventually stabilizing. This trend is consistent with previous research reports.

Currently, there are still some challenges to the widespread adoption of MAKO robot-assisted TKA surgery worldwide. On the one hand, the surgical robot is expensive, has certain requirements for the operating room, involves complex installation and debugging, and requires regular maintenance, which is costly; on the other hand, the Mako robot-assisted TKA surgery requires a certain amount of surgical volume upfront in terms of the learning curve and the steps of pre-osteotomy registration, preparation, and intraoperative debugging. In summary, TKA assisted by the MAKO robot demonstrates superior short-term clinical efficacy compared to traditional manual total knee arthroplasty. The MAKO robot-assisted technology facilitates accurate prosthetic placement and planned limb alignment with good safety.

## Conclusion

Compared with conventional manual total knee arthroplasty, MAKO robot-assisted TKA demonstrates better short-term clinical efficacy, achieves better alignment planning, and maintains good safety.

## Limitations

This study is a single-centre retrospective study with a relatively small sample size, which may introduce bias to the reliability of the study results. Additionally, the follow-up results in this study are all from short follow-ups, lacking long-term follow-up data. Therefore, future large-sample, multicenter, long-term retrospective studies are needed to enhance the credibility of the research.

## Data Availability

All data included in this study are available upon request by contact with the corresponding author on reasonable request.
